# Hereditary Sensory and Autonomic Neuropathy Type 2: A Case Report and a Review of the Literature

**DOI:** 10.3390/brainsci15111163

**Published:** 2025-10-29

**Authors:** Cosmanna Ragucci, Alessandro Furia, Flavia Palombo, Maria Pia Giannoccaro, Veria Vacchiano, Alex Incensi, Vitantonio Di Stasi, Giovanni Rizzo, Rocco Liguori, Vincenzo Angelo Donadio

**Affiliations:** 1 Dipartimento di Scienze Biomediche e Neuromotorie, Università di Bologna, 40138 Bologna, Italyvincenzo.donadio@unibo.it (V.A.D.); 2 IRCCS Istituto delle Scienze Neurologiche di Bologna, UOC Clinica Neurologica, 40139 Bologna, Italy; 3 IRCCS Istituto delle Scienze Neurologiche di Bologna, Programma di Neurogenetica, 40139 Bologna, Italy

**Keywords:** hereditary sensory and autonomic neuropathy type 2A, *WNK1/HSN2* gene

## Abstract

We report a case of hereditary sensory and autonomic neuropathy presenting with childhood-onset symmetric distally predominant limb hypoesthesia to tactile, thermal, and painful stimuli. Exome sequencing identified a homozygous pathogenic variant in the with-no-lysine (K) kinase 1 (*WNK1*), lysine deficient protein kinase 1 gene. The clinical, electrophysiological, and genetic findings confirmed a diagnosis of hereditary sensory and autonomic neuropathy type 2A (HSAN 2A). This case highlights the importance of genetic confirmation in the evaluation of early-onset neuropathies, especially when the most common causes have been ruled out. Significantly, our observations underscore the potential role of skin biopsy in identifying autonomic abnormalities in HSAN 2, possibly contributing to a better understanding of these rare neuropathies. We also reviewed the reported cases of this disease in the literature to highlight its phenotypic variability.

## 1. Introduction

Hereditary sensory and autonomic neuropathies (HSANs) encompass rare clinically and genetically heterogeneous disorders of the peripheral nervous system [1,2]. The pathology is mainly defined by degeneration of sensory and autonomic neurons and axonal changes [1,2,3], with variable involvement of unmyelinated as well as small and large myelinated peripheral nerve fibers [4]. Clinically, sensory disturbances are the predominant features, characterized by slowly progressive multimodal sensory loss [4] with a variable degree of autonomic and motor involvement [1]. HSANs are mostly associated with autosomal recessive (AR) transmission; sporadic cases have also been reported [5,6].

HSANs were historically grouped into five subtypes (I–V), as proposed by Dyck and Otah [3], according to the age at presentation, core phenotypic features, and genetic transmission [1,2,7,8]. Nevertheless, new molecular correlations are still being defined, and the classification of emerging entities remains a source of debate. Indeed, approximately 20 genes have been associated with HSAN cases [9].

In the past decade, more entities have been added to the original classification, thus defining eight subtypes [4,10,11]. Pathogenic variants in *TECPR2* have been reported as causative of HSAN 9, primarily known as hereditary spastic paraplegia 49 (HSP 49) [12,13].

HSAN 1 is an autosomal dominant (AD) form that has been described as the most frequent HSAN subtype [4,6] and is caused by pathogenic variants in *SPTLC1*, *SPTLC2*, *RAB7A*, *ATL1*, *DNMT1* genes [2,4]. It is characterized by a predominantly sensory phenotype with adult onset in the second to fourth decade of life, presenting with painless distal ulcerations and mutilating arthropathies due to impaired nocipeption and temperature perception, in conjunction with lancinating and burning pains [4]. Distal weakness and atrophy have also been described in HSAN 1, features that may mimic other hereditary sensory and motor neuropathies [2]. Among the AR forms, HSAN 3 (also known as familial Dysautonomia, previously Riely–Day syndrome) is the most extensively investigated category and is often considered the prototype for comparison with other HSAN subtypes [14,15]. It occurs predominantly in individuals of Ashkenazi Jewish ancestry and is caused by pathogenic variants in the *IKBKAP/ELP1* gene [4,14,15]. The disorder typically presents at birth and is pathologically characterized by progressive depletion of unmyelinated and thinly myelinated sensory and autonomic peripheral nerve fibers, resulting in pervasive autonomic dysfunction and sensory neuropathy [4,14]. Early manifestations include motor incoordination, hypotonia, feeding difficulties and aspiration due to oropharyngeal incoordination, breath holding spells and insensitivity to hypoxemia, leading to delayed motor development [4,14,16]. Lower limb sensory neuropathy predominantly affects small fibers and leads to Charcot joints and aseptic necrosis [4]. Preserved visceral sensitivity to pain, profound cardiovascular dysautonomia, and recurrent dysautonomic crises are key clinical features [4]. The latter manifest with gastrointestinal symptoms (nausea, vomiting) as well as cardiovascular dysregulation (hypertension, tachycardia), and may include hyperhidrosis, skin blotching, and increased respiratory and gastrointestinal secretions [4,14,16]. The disorder is also associated with central nervous system manifestations, including emotional lability—exacerbated during autonomic crises—and impaired executive and organizational skills, although intellectual disability is not observed [4,14]. Seizures may result from hypoxia and electrolytic disturbances [14]. HSAN 4 (congenital insensitivity to pain with anhidrosis) is another distinct AR form associated with biallelic variants in *NTRK1* gene [1]. The clinical onset is at birth, and, similarly to HSAN 3 [14], it is regarded as a neurodevelopmental disorder [15]. The condition is frequently associated with consanguinity (50%) and has been reported in patients of Mediterranean, Middle Eastern, Japanese, Indian, and Pakistani ancestry [15]. The disease presents both sensory and autonomic features, including widespread decreased nociception that also affects visceral sensation and cranial nerves, often resulting in self-mutilation [15]. Generalized anhidrosis is a hallmark feature, more frequently involving the trunk and upper extremities due to impaired thoracolumbar sympathetic outflow, while other autonomic disturbances are usually mild to absent [15]. Additional distinctive clinical features include febrile peaks and impaired healing of ectodermal structures. Neuropathological studies demonstrate predominant involvement of unmyelinated neurons [15]. HSAN 5 (congenital insensitivity to pain with partial anhidrosis), first described in the Swedish population [4], is associated with biallelic pathogenic variants in the *NGFB* gene [2], with rare cases exhibiting mutations in *NTRK1*, suggesting that HSANs 4 and 5 may be allelic conditions [17]. HSAN 5 presents milder anhidrosis and preserved cognitive functions, in contrast to HSAN 4 [1]. The disease presents at birth with congenital insensitivity to pain leading to joint deformities, resulting from pathological involvement of unmyelinated and small myelinated fibers [2]. HSAN 6 has AR inheritance and is associated with *DST* gene mutations. It was initially described in an Ashkenazi Jewish family and shows significant clinical overlap with HSAN 3 [18]. HSAN types 7 and 8 are indicated as congenital insensitivity to pain (CIP2 and CIP3), associated with AD and AR inheritance, respectively [4]. HSAN 7 is associated with mutations in the *SCN11A* gene which encodes a voltage-gated sodium channel, while HSAN 8 is caused by mutations in the *PRDM12* gene [4]. Both forms present loss of pain and temperature sensation and sweating abnormalities [4]. Of note, HSAN 7 share some clinical features with HSAN 2D, which is caused by mutations in *SCN9A* [4].

HSAN 2 (also known as congenital sensory neuropathy, “acrodystrophic neuropathy” [19] or Morvan’s disease) is a rare entity characterized by degeneration of peripheral sensory and autonomic neurons, with predominant involvement of large and small myelinated fibers and, to a lesser extent, unmyelinated fibers [16]. The prevalence of the disease is unknown, with only a few hundred affected individuals reported worldwide [20]. HSAN 2 is genetically heterogeneous, associated with biallelic pathogenic variants in *KIF1A*, *RETREG1 (FAM134B)*, *SCN9A*, or *WNK1* [20] genes, with AR pattern of inheritance, although sporadic cases have been described [16]. *WNK1*-related HSAN 2 is referred to as HSAN 2A [20]. From a clinical perspective, HSAN 2 subtypes exhibit highly similar phenotypic presentations, with sensory deficits being the predominant feature [2,20]. Cases have been reported across several groups, without any known ethnic predilection [16]. However, sporadic familial and regional clusters have been described, particularly in the French Canadian population [21], where founder mutations have been reported [22]. Onset usually occurs in infancy or early childhood [2], with a slowly progressive or non-progressive clinical course [16]. The pathophysiology is characterized by early impairment of large and small myelinated sensory fibers [16]. Sural nerve biopsies typically reveal an almost complete depletion of myelinated nerve fibers, whereas unmyelinated fibers may be preserved, albeit moderately reduced in number [3,16,19].

Initial manifestations typically include distal limb numbness in a stocking-and-glove distribution, followed by progressive impairment of pain, temperature, and touch sensation [2]. The disorder usually leads to a profound loss of all sensory modalities, including nociception, that predominantly affect distal regions [23]. Autonomic involvement is variable and has been described as a possible early manifestation, although generally milder than in other HSAN subtypes (e.g., HSAN 3) [16]. Autonomic manifestations may include episodic and localized hyperhidrosis or anhidrosis, delayed onset of overflow lacrimation, gastroesophageal reflux, esophageal and intestinal dysmotility, and heightened pupillary sensitivity to parasympathomimetic agents [4,16]. Moreover, hypogeusia may result from hypotrophic fungiform papillae [16], as observed in HSAN 3 [14,15].

Clinically, neonatal presentation includes generalized hypotonia with motor incoordination, profound loss of deep sensory modalities, severe feeding difficulties with dysphagia, and weak gag reflex and frequent apnea [16]. Sensory deficits may contribute to delayed developmental milestones, scoliosis, and neuropathic remodeling, with Charcot joints and skeletal dysplasia [4,16]. Pain insensitivity leads to recurrent unnoticed injuries, which may progress to osteomyelitis or necessitate amputations [4]. Additional features may include varying degrees of intellectual disability and sensorineural hearing loss (SNHL), while motor function is generally preserved [4,16]. Self-mutilation associated with the first dentition has been described [16]. The neurological examination usually discloses marked sensory abnormalities and reduced or absent tendon reflexes, whereas other neurological functions are typically preserved [16].

Nerve conduction studies (NCS) usually demonstrate reduced or absent sensory nerve action potentials, with normal or mildly affected motor nerve conduction velocities and variable degrees of compound motor action potential involvement [20,23].

The literature is limited due to the rarity of the disease, ranging from single-case descriptions to small case series. Poor understanding of the pathophysiology of these disorders contributes to diagnostic delays and morbidity due to osteoarticular complications. The clinical management of HSAN 2 is supportive and preventative [16], with early instructions on foot care [24].

Here we reported a new case of HSAN type 2 and reviewed the scientific literature of reported cases to highlight its phenotypic variability.

## 2. Case Report

The case of a patient diagnosed with HSAN type 2A is described.

The clinical history is detailed as follows:

Demographics: 33-year-old male of Sicilian origin, born to non-consanguineous parents.

Family history: negative for neurological disorders.

Medical history:

- Previously healthy, the patient reported no issues during the neonatal period or with psychomotor development.
- Childhood-onset of symmetric distally predominant lower limb tactile, thermal, and pain hypo-anesthesia and tactile, thermal and pain hypoesthesia of the palms of the hands, with history of frequent burns and injuries especially to the lower extremities. He additionally described clumsiness in fine hands movements, particularly in the absence of visual feedback. Moreover, the subject reported dryness of the palms of the hands and soles of the feet, without sweating alterations and occasional gait instability. He did not experience positive sensory symptoms.
- At the age of 25 the patient underwent amputation of the second toe of the left foot due to a cutaneous ulcer secondary to unrecognized traumas, which progressed to deep dermal infection and osteomyelitis. At the age of 32 the patient was diagnosed with squamous cell carcinoma of the third toe of the left foot, arising from a cutaneous ulcer, followed by amputation of the distal phalanx of the third toe of the left foot.
- Previous electrophysiologic evaluation demonstrated sensitive axonal polyneuropathy. Previous genetic testing in *MFN2*, *NEFL*, and *GDAP1* genes, associated with Charcot–Marie–Tooth neuropathy type 2 (CMT2) was negative.

The patient was referred to our Clinic for diagnostic evaluation.

The neurological examination showed tactile and thermal-pain hypoesthesia of the hands and lower limbs and hypo-pallesthesia with distally predominant gradient, absent proprioception, and upper limb sensory ataxia. Deep tendon reflexes were absent with mute plantar reflex and multidirectional oscillations during the Romberg test.

**Figure 1:**
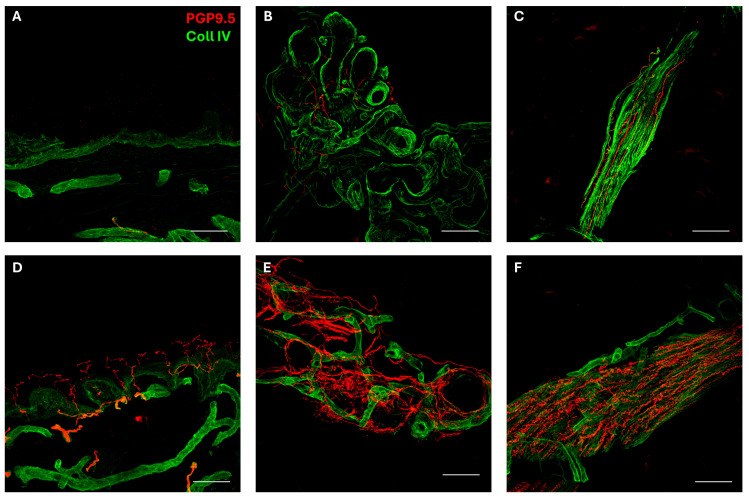
Somatic and autonomic innervation in the patient and a healthy control subject. Epidermal and autonomic innervation disclosed by confocal microscope (×40) in the patient with HSAN 2A (**A**–**C**) and an age-matched control subject (**D**–**F**). Nerve fibers are marked in red (PGP 9.5 staining), whereas the collagen staining is shown in green. Free-ending PGP9.5 immunoreactive nociceptive fibers are evident in the epidermis of the control (**D**). The basement membrane separating epidermis from dermis is marked by collagen staining. PGP 9.5 positive fibers were absent in the patient (**A**). Bar corresponds to 100 microns. Autonomic PGP9.5-positive nerve fibers encircle sweat tubules (**E**) and innervate muscle arrector pilorum (**F**) in the healthy control, but they were markedly reduced in the patient (**B**,**C**). Bar corresponds to 50 microns.

The patient underwent the following:

- Blood tests including autoimmune and microbiological screening, anti-neuronal antibodies, anti-ganglioside antibodies IgG and IgM, and autoimmune encephalitis panel, all of which were unremarkable.
- Electromyography (EMG) with nerve conduction studies (NCS), which confirmed a severe predominantly sensitive polyneuropathy with non-elicitable sensory nerve action potential (SNAP) in the median, ulnar, and sural nerves using near-nerve technique. Despite a 24–28% reduction in motor conduction velocity of the right median and ulnar nerves, the compound muscle action potential (CMAP) amplitudes remained within normal limits, indicating preserved motor axon integrity. F-waves of the median and ulnar nerves, as well as bilateral tibial nerves, showed normal latency and persistence.
- Skin biopsy was performed according to a previously described method [25,26]. As shown in Figure 1, the biopsy disclosed a severe autonomic small-fiber neuropathy involving both sweat glands and muscle arrector pilorum compared to normal innervation [27]. In addition, epidermal somatic fibers were absent both in the proximal and distal skin sites of the patient [28].
- The evaluation of autonomic control of cardiovascular reflexes reported normal cardiovagal modulation and sympathetic responses, excluding orthostatic hypotension.
- The exome sequencing identified the presence of the homozygous pathogenic variant c.3526_3529del_p.Thr1176CysfsTer21 (NM_213655.5) in the *WNK1* gene, already reported [29]. Biallelic pathogenic variants of the *WNK1* gene (OMIM*605232) are associated with HSAN 2A. Thus, the result was considered consistent with the clinical picture.

Refer to the Supplementary Materials section for a comprehensive overview of the conducted assessments.

## 3. Discussion

The genetic cause of HSAN 2A was first identified by Lafreniere et al., who reported two founder mutations (HSN2 ORF c.594delA and c.918_919insA) in the *WNK1/HSN2* gene at 12p13.33 in five families of affected individuals from the two known population clusters of this disease in rural Quebec, Nova Scotia, and Newfoundland [21]. Thereafter, two new founder mutations (HSN2 ORF c.943C > T and c.918_919insA) were identified in a large cluster of 13 French Canadian families from the Lanaudière region in Southern Quebec, with one Canadian subject from Lebanon [22]. These patients exhibited a rather homogeneous slowly progressive phenotype, with symptom onset in the first decade of life and leg amputations from the second decade.

The first identification of the disease associated with a new *WNK1/HSN2* variant outside the original disease clusters occurred in a Lebanese family [30]. Subsequently, most reports in the literature have described novel variants in isolated cases or individual families, and systematic descriptions of large patient cohorts belonging to different populations are lacking.

To date, only one further instance of a recurrent founder-effect mutation has been identified outside the French Canadian population, namely in five patients belonging to a cohort of thirty-three unrelated patients from Japan (NM_001184985 c.3237_3238insT) [9]. Four of five patients reported the onset of insensitivity to pain in infancy, while one case reported hyperidrosis from the age of 17 years. All the subjects experienced some degree of autonomic involvement (orthostatic hypotension, dyshidrosis, dysuria). Of note, two cases developed positive sensory symptoms at adult age, with Case I referring episodic “electric shock-like” pain and “piercing” pain in multiple joints and Case III experiencing unilateral upper limb “electric shock-like” pain. Moreover, burning acroparesthesias were also reported by two siblings in a Han Chinese family, who ultimately manifested acro-osteolysis [31]. The disease has been further reported in East Asia, with the variant NM_001184985 c.3237_3238insT documented in two more reports from Japan [32,33]. The variant has been described in homozygosity in one patient with a delayed manifestation in teenage years of hyperhidrosis and chilblain-like edema of the extremities. Remarkably, no histopathological abnormalities consistent with autonomic dysfunction were found after skin biopsy [33].

Notably, four patients from two unrelated families in Chiapas, Mexico, carrying the same variant (HSN2 ORF c.1219_1226delTCTCAGCA), exhibited high intrafamilial variability in disease onset, with one case possibly showing a late onset compared to two affected siblings [34]. In addition, an Iranian family with four affected members carrying the c.3718C > A (NM_213655.5) variant showed some degree of phenotypic heterogeneity with disease onset ranging from 6 months to 10 years [35]; however, all patients displayed non-progressive symmetrical multimodal sensory loss leading to distal mutilations. Furthermore, milder phenotypes without deformities or amputations as well as delayed presentations are possible, as recently reported in two offspring of Punjabi descent from Pakistan (NM_213655.4: c.3464delinsAC) [36].

Cases of the disease have also been documented in Europe. Thus far, pathogenic variants have been identified in three unrelated European patients—from Italy, Austria and Belgium—heterozygous for c.254delC and c.1089_1090insT, homozygous for c.550C > T, homozygous for c.1064_1065delTC, respectively (HSN2 ORF)—with a variable onset from early infancy to childhood of sensory loss leading to distal ulcers and necrosis [37]. Moreover, a single case was recently reported in Poland, homozygous for c.2897_2898delAG (NM_213655.3), with the onset of dysphagia in infancy and a later manifestation of nociception loss, resulting in injuries, ulcerations, and toe dysplasia [38]. Of note, this patient did not report autonomic dysfunction, whereas the sympathetic skin response result was markedly abnormal. Another single case reported in Belgium, heterozygous for c.718A>T and c.1192_1196del (HSN2 ORF) [39], showed an intact vibration sense with selective involvement of nociception, thermoception, and touch sensation in stocking and glove distribution.

The literature review results are summarized in Table 1. All the reported variants have been renumbered with reference to NM_213655.5 (ENST00000340908.9), which is the longest neuron-specific transcript containing HSN2 (11,552 bp).

**Figure 2:**
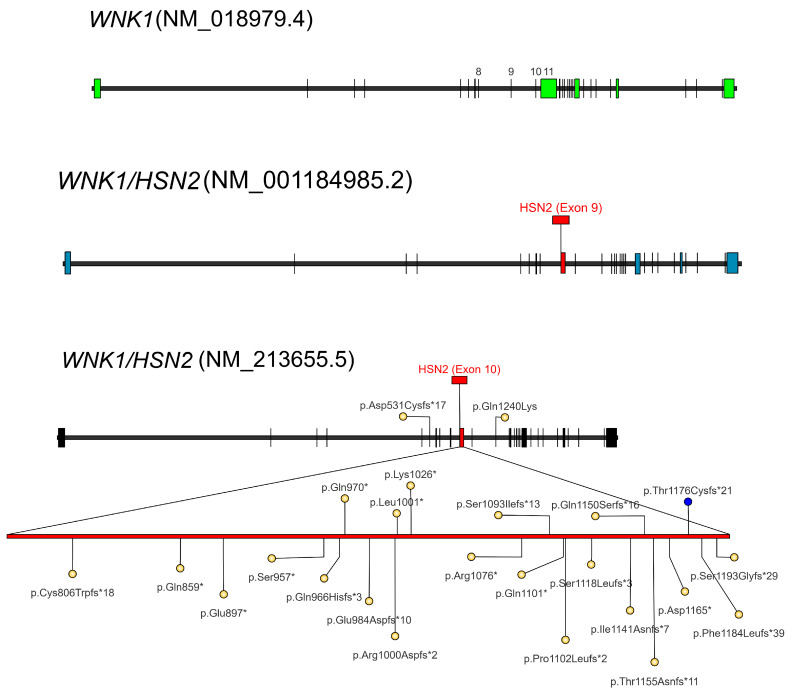
Schematic representation of the *WNK1/HSN2* gene structure. To allow transcript comparison, the “Canonical” isoform is presented (Q9H4A3-1; RefSeq NM_018979.4), which does not include the *HSN2* exon. *HSN2* is a nervous system-specific exon (indicated in red) included in isoform 5 (Q9H4A3-6, “Dorsal root ganglia and sciatic nerve variant”; RefSeq NM_001184985.2) and isoform 4 (Q9H4A3-5, “Brain and spinal cord variant”; RefSeq NM_213655.5). The locations of the identified variants are numbered with reference to transcript NM_213655.5. Previously reported variants are represented by yellow filled circles, while the variant identified in this study is highlighted in blue [45].

*WNK1* is a large and complex gene that includes 33 exons and is ubiquitously expressed. This gene is alternatively spliced in a tissue-specific manner, giving rise to multiple isoforms (Figure 2). In particular, HSN2 is an alternatively spliced large exon expressed in neuronal tissues and during development. This exon is located in intron 9 with respect to the Long (Canonical) transcript (NM_018979.4) and is expressed in the neural isoforms (NM_213655.5 and NM_001184985.2). The majority of HSAN 2A-associated variants cluster within the HSN2 exon, as shown in Figure 2.

Inherited neuropathies characterized by pain insensitivity result from genetic pathogenic variants that disrupt normal signaling or lead to the degeneration of sensory afferent neurons located in the dorsal root ganglia (DRG), which are critical for the detection and transmission of noxious stimuli [23]. HSAN 2A is caused by loss-of-function of the *WNK1* gene, and pathogenic mutations have been identified within the large alternatively spliced exon known as “HSN2”, whose expression is specific to nervous tissues [23]. HSN2 encodes a 498-amino-acid domain located downstream of the protein kinase domain [23]. To date, the vast majority of the recessive variants are nonsense or frameshift mutations in exon 10 [2] (Table 1 and Figure 2), with premature truncation of the WNK1/HSN2 nervous system-specific protein [29,42], resulting in proteins that lack the C-terminal domains [23]. In one case, an exon 10 variant has been reported combined in the heterozygous state with a large deletion containing *WNK1* (copy number decrease) [31]. In two cases, variants in exon 10 were found in compound heterozygosity with concurrent variants in exon 6 [42] and exon 12 [35].

The underlying molecular mechanism by which *WNK1/HSN2* mutations give rise to a pain-insensitive phenotype remains to be fully elucidated. According to previous evidence, HSN2 is ubiquitous in DRG neurons, although most strongly expressed in large proprioceptive neurons and at lower levels in small-diameter nociceptive C-fibers [23]. As a matter of fact, an immunohistochemical study on murine models demonstrated that WNK1/HSN2 expression was significantly stronger in dorsal roots containing sensory axons than in ventral roots [42], consistently with clinical motor function preservation. Interestingly, WNK1/HSN2 expression was undetectable in axonal nerve fibers, whereas the surrounding Schwann cells were positive [42]. However, this finding has not been replicated in human DRG expression analyses, where satellite Schwann cells were positive for WNK1 but did not show strong HSN2 expression [23].

We found a severe sensory axonal polyneuropathy on NCS, associated with a 24–28% reduction in motor conduction velocity of the median and ulnar nerves. The percentages represented the degree of deviation from the corresponding age-adjusted mean, using the lower 95% confidence limit of our laboratory’s normative data as a reference threshold to detect abnormality. As already mentioned, in HSAN 2 motor nerve conduction velocities and compound motor action potentials are usually normal or only slightly below the lower limit of normal [3,16,19], reflecting indeed a variable degree of motor involvement [16]. However, the observed abnormality did not meet the criteria for acquired demyelination [46] and was not suggestive of inherited demyelination [24]. Importantly, no temporal dispersion was observed in motor responses or F-waves, ruling out a demyelinating process. Indeed, our NCS documented a variable axonal involvement of the examined sensory nerves. After applying a consistent and reproducible sensory antidromic technique with surface electrodes, sensory responses were recordable in some nerves (e.g., the left median and the left ulnar nerves) but absent in others (e.g., the right ulnar and the sural nerves), reflecting a non-uniform neuropathic involvement. A more sensitive method, the orthodromic near-nerve technique using needle electrodes, confirmed the absence of recordable responses in the sural nerves bilaterally and in the right median nerve, further supporting significant axonal loss (see NCS in the Supplementary Material).

Although the NCS findings are consistent with primary sensory axonopathy, a concomitant Schwann cell involvement in the pathogenesis and in the observed electrophysiologic abnormalities remains intriguing. For instance, the myelin basic protein (MBP) is known as a WNK1 substrate [23,47], and aberrant myelination has been described as a possible feature of HSAN 2 associated with reduced conduction velocity [23]. An alternative speculative hypothesis suggests that WNK1/HSN2 may instead influence the response to nerve damage by regulating Schwann cell proliferation through a MEKK2/3-dependent mechanism, as observed in other tissues [42].

However, WNK1 plays a role in regulating sodium and chloride ion flux as well as membrane excitability [20]. WNK1 also influences the expression of TRPV4, a cation channel involved in nociceptive signaling, thereby functioning as a key modulator of pain perception in peripheral nerve endings [20]. Accordingly, a functional impairment of axonal transmission could underlie the sensory loss, including nociceptive signaling.

The identified variant, NM_213655.5:c.3526_3529del (p.Thr1176Cysfs*21) in the *WNK1/HSN2* gene, is classified as pathogenic in major genetic databases. The variant has been already described in previous reports in subject belonging to two families of Maltese descent with the congenital form and presenting with distal ulcers, amputations, and osteonecrosis [29]. Based on previously reported data, no definitive conclusions can be drawn regarding genotype–phenotype correlations for *WNK1/HSN2* variants. As summarized in Table 1, considerable clinical variability exists even among patients carrying identical variants. Nonetheless, the identification of novel variants of the same gene in isolated cases from diverse populations underscores the genetic homogeneity of this rare disorder, as previously stated [37,44].

We reported a case of HSAN 2A associated with a known pathogenic biallelic nonsense variant in the *WNK1* gene, inducing a loss-of-function effect. This variant creates a premature translational stop signal, with the production of a truncated product with no expected residual function, as previously described [29]. Our patient’s clinical presentation was milder with respect to symptom severity and age at onset compared to previously reported cases, although autonomic involvement was not explicitly addressed by Davidson et al. [29]. Therefore, this case broadens the current understanding of phenotypic variability among individuals carrying the same variant.

Notably, we reported histopathological findings of widespread somatic and autonomic small-fiber neuropathy, with neither concomitant cardiovascular autonomic dysfunction nor overt autonomic symptomatology. Our patient experienced dryness of the palms and soles, similarly to the case previously reported in Korea, which otherwise presented with a more severe clinical phenotype, including the amputation of both lower legs and several fingers [43]. Both cases exhibited normal autonomic function tests, including the Valsalva maneuver, the tilt test, and deep breathing. However, in the Korean case, the sympathetic skin response was additionally assessed and was found to be normal. Consistently, sural nerve biopsy showed preservation of unmyelinated nerve fibers, with a severe loss of large and small myelinated nerve fibers. Despite a similar clinical presentation, our patient showed histopathological evidence of a severe autonomic small-fiber neuropathy.

Skin biopsy is more commonly employed in conditions such as HSAN 4, in which sural nerve biopsy shows a complete loss of unmyelinated fibers, with preservation of myelinated nerve fibers [3]. Accordingly, skin biopsy demonstrates a deficit of epidermal C and Aδ fibers, associated with the absence or hypoplasia of dermal sweat glands lacking innervation [16,48,49,50,51]. Conversely, skin biopsy application in HSAN 2 is not routinely considered. Our findings suggest that this technique might offer additional diagnostic value in HSAN 2 by demonstrating pathological involvement of unmyelinated autonomic fibers.

Indeed, orthostatic hypotension is not a typical finding in HSAN 2, whereas it is more commonly described in HSAN 3, which, in contrast, is characterized by prominent autonomic dysfunction presenting as orthostatic hypotension, loss of compensatory tachycardia, supine hypertension, and dysautonomic crises [14,16]. As a consequence, it is not surprising that we did not find alterations on autonomic testing, also considering the absence of consistent autonomic cardiovascular symptoms. However, as described in a previous study by Donadio et al. [27], which compared the diagnostic accuracy of skin biopsy for detecting sympathetic nerve abnormalities in patients with peripheral autonomic neuropathy, cardiovascular reflexes and skin sympathetic responses were found to be less sensitive. As it has been previously speculated, routine autonomic tests are indirect assessments of functions which may be influenced by the functional state of target organs; moreover, the dual sympathetic and parasympathetic outflow to the heart may compensate for selective dysfunctions of one branch [27]. In this setting, skin biopsy confirms its role as a sensitive technique for evaluating peripheral autonomic function. As previously noted, this methodology traditionally plays a major role in detecting abnormalities in unmyelinated fibers [52], which are relatively preserved compared to the marked loss of myelinated fibers in HSAN 2 [3]. We could hypothesize that autonomic alterations detected by skin biopsy may represent an early stage in the development of autonomic neuropathy, an idea also proposed for somatic fibers [53]. However, this speculation is not fully supported by the typically non-progressive clinical course of HSAN 2 after childhood [16], even though autonomic worsening (e.g., with later onset of urinary dysfunction) has been described [20]. In this regard, further systematic investigation of these issues in larger patient cohorts would be of interest, as well as longitudinal assessments to monitor the possible appearance of new autonomic symptoms over time.

## 4. Conclusions

We identified a genetic variant which has been previously described in the literature, further supporting the genetic homogeneity of this rare disorder. Molecular studies and identification of new genetic correlations for HSAN are ongoing. Extensive genetic testing, including whole-exome sequencing, is essential for identifying rare genetic neuropathies, especially in cases when targeted genetic analyses have been inconclusive. To our knowledge, skin biopsy is not routinely included in the diagnostic workup of HSAN 2, due to the milder involvement of unmyelinated fibers. As in our case, it may be a valuable tool to detect the subclinical pathological involvement of somatic and autonomic fibers, demonstrating that the involvement of this system may be present even in the absence of clinical manifestations and is therefore likely underestimated.

## Figures and Tables

**Table 1:** Literature Review.

Population [Reference] Family/Patient	*WNK1/HSN2* Variant (NM_213655.5) [Type of Mutation]	Onset Age (Years)	First Symptom/Clinical Presentation	Autonomic Involvement	NCS
China Ma S. et al., 2025 [40] A single case	Homozygous c.2689G>T; p.(Glu897*) [Nonsense]	12	Bilateral toe ulceration and infections	None	Severe sensory nerve damage
Turkey Naghinejad M. et al., 2024 [41] 3 offspring of Azari Turkish descent	Homozygous c.3226C>T; p.Arg1076* [Nonsense]	I—Childhood II—U III—8	I, II, III—Dysphagia, hypoesthesia and recurrent distal wounds, self-mutilating behavior, hyperkeratosis. III—Distal paresthesia	I, II—Constipation III—None	I—Generalized axonal sensory neuropathy II, III—Mild nerve and muscle involvement
Pakistan Pastore et al., 2020 [36] 2 offspring of Punjabi Pakistan descent	Homozygous c.3463dup; p.Thr1155Asnfs*11 [Frameshift]	I—Puberal age II—U	I—Deformities II—U, milder phenotype	None	U
China Wang et al., 2019 [31] 2 siblings in a Han family	Heterozygous c.3002T>G; p.Leu1001* [Nonsense] c.3352del; p.Ser1118Leufs*3 [Frameshift]	Infancy	Analgesia, ulcers and neurogenic osteolysis, burning acroparesthesias.	Sweating disorders	U
Iran Rahmani et al., 2018 [35] 4 affected siblings	Homozygous c.3718C>A; p.Gln1240Lys [Nonsense]	I—6 months II—2 III—7 IV—10	Distal limb multimodal reduced sensory function with amputations	None	III, IV –Symmetric peripheral sensory axonal neuropathy
Japan Shima et al., 2018 [33] A single case	Homozygous c.3492dup; p.Asp1165* [Frameshift]	17	Autonomic symptoms with fingers and toes ulceration	Hyperidrosis and extremities chilblain like edema	U
Japan Yuan et al., 2017 [9] 33 unrelated patients	Homozygous c.3492dup; p.Asp1165* [Frameshift] Heterozygous c.2870C>G; p.Ser957* in Case II [Nonsense]	I, II, III, V—Infancy IV—17	I, II, III, V—Analgesia IV—Hyperhidrosis	I—OH, TF, defecation disorder II—Dyshidrosis, urination disorder III, IV, V—Dyshidrosis	SNAPs could not be evoked, markedly reduced in Case IV
Japan Yamada et al., 2016 [32] A single case	Homozygous c.3492dup; p.Asp1165* [Frameshift]	Infancy	Multimodal sensory loss and taste disorder	OH, fluctuation in body temperature, and absence of defecatory urge	Absent SNAPs of the median, ulnar, and sural nerves.
Belgium De Filette et al., 2016 [39] A single case	Compound heterozygous c.3550_3554del; p.Phe1184Leufs*39 [Nonsense] c.3076A>T | p.Lys1026* [Frameshift]	3	Ecchymoses of the toes	OH, GERD, hand hyperhidrosis with cold-triggered purple discoloration	Absent SNAPs in upper and lower limbs
East Europe, Poland Potulska-Chromik A. et al., 2012 [38] A single case	Homozygous c.2898_2899del; p.Gln966Hisfs*3 [Frameshift]	1	Dysphagia and loss of nociception	None	Absent SNAPs
Malta Davidson et al., 2012 [29] 2 unrelated cases	Homozygous c.3526_3529del; p.Thr1176Cysfs*21 ^#^ [Frameshift] Compound heterozygous c.2418_2419del; p.Cys806Trpfs*18 [Frameshift]	Congenital	Ulcers, distal amputations	None	Sensory motor axonal neuropathy
Chiapas, Southeast of Mexico Pacheco-Cuellar G. et al., 2011 [34] 4 patients belonging to 2 families	Homozygous c.3577_3584del; p.Ser1193Glyfs*29 [Frameshift]	I—19 II—20 III—10 IV—9	Sensory loss, osteolysis and Charcot joints, amputations	None	I and IV—U II and III—absent SNAPs
France Shekarabi M. et al., 2008 [42] A single case	Compound heterozygous c.2998del; p.Arg1000Aspfs*2 [Frameshift] c.1591_1592del; p.Asp531Cysfs*17 [Frameshift]	U	U	Hand hyperhidrosis	U
Korea Cho H.J. et al., 2006 [43] A single case	Compound heterozygous c.3492dup; p.Asp1165* c.2575C>T; p.Gln859* [Nonsense]	11	Multimodal limb sensory loss	Dry hands	Distal sensory dominant poly neuropathy
Japan Takagi M. et al., 2006 [44] A single case	Homozygous c.3492dup; p.Asp1165* [Frameshift]	Teenage years.	Pain insensitivity	None	Absence of SNAPs in the median and sural nerves of both sides
Europe (Italy, Austria, and Belgium) Coen K. et al., 2006 [37] 3 unrelated families (CMT-451, CMT-260, and CMT-178)	Family CMT-451 Patient II-2: compound heterozygous c.2612del; p.Pro871Hisfs*14 [Frameshift] c.3447dup; p.Gln1150Serfs*16 [Frameshift]. Family CMT-260 Patient II-6: Homozygous c.2908C>T; p.Gln970* [Nonsense]. Family CMT-178 Patient III-1: Homozygous c.3422_3423del; p.Ile1141Asnfs*7 [Frameshift]	II-2—6 months II-6—early childhood III-1—2	II-2: Difficulties in hand manipulation II-6: Clumsiness of the hands, recurrent osteomyelitis. III-1: Poor wound healing and recurrent hand and foot ulcers	None	III-1: Sensory neuropathy with absent SNAPs in all limbs
Quebec, Newfoundland and Nova Scotia Lafreniere R.G. et al., 2004 [21] Five families from the two population clusters in Canada: Newfoundland F1 (8 A), F2 (2 A); French Canada F3 (2 A), F4 (1 A); Nova Scotia F5 (2 A)	Patient F1-70 from Newfoundland: Homozygous c.2952del; p.Glu984Aspfs*10 [Frameshift]; Patient F5-301 from Nova Scotia: Homozygous c.3276dup; p.Ser1093Ilefs*13 [Frameshift].	Early childhood	Reduced nociception and cold-induced numbness in hands and feet	None	U
French Canadian from Southern Quebec (Lanaudière region) Roddier et al., 2005 [22] 18 patients belonging to 13 families and one Canadian patients of Lebanese origin	Mutation 1: c.3301C>T; p.Gln1101* [Nonsense] Mutation 2: c.3276dup; p.Ser1093Ilefs*13 [Frameshift] 56% Homozygous c.3301C>T 6% Homozygous c.3276dup 38% Compound heterozygotes The Canadian child of Lebanese origin resulted homozygote for a novel mutation: c.3226C>T | p.Arg1076* [Nonsense].	Infancy or early childhood	Paronychia, ulcers and Charcot joints with multimodal sensory loss	Minimal dysautonomia (U)	Absence of SNAPs
Lebanon Rivière J.B. et al., 2004 [30] A family with 4 affected individuals	Homozygous c.3305del; p.Pro1102Leufs*2 [Frameshift]	First decade	Loss of sensation and insensitivity to pain causing ulcers and amputations	U	U

Keys: OH: orthostatic hypotension; GERD: gastroesophageal reflux disease; TF: thermoregulatory failure; SNAPs: sensory nerve action potentials; U: unspecified; ^#^ present study. All variant nomenclature adheres to the guidelines of the Human Genome Variation Society (HGVS). Variants have been renumbered with reference to NM_213655.5. Patients are identified by sequential Roman numerals; original numeric codes have been preserved where specified in the original publication. The onset symptoms are reported; if unavailable, the clinical presentation at initial evaluation is indicated. Note: all the reported variants are loss-of-function.

## Data Availability

The original contributions presented in this study are included in the article/Supplementary Material. Further inquiries can be directed to the corresponding author.

## Supplementary Materials

The following supporting information can be downloaded at https://www.mdpi.com/article/10.3390/brainsci15111163/s1, Table S1: Laboratory investigations; Table S2: In silico genes panel analysis for hereditary neuropathies panel; Table S3: Histopathological investigations: Protocol and Results; Table S4: Neurophysiological Investigations: Motor Nerves; Table S5: Neurophysiological Investigations: Sensory Nerves; Table S6: Neurophysiological Investigations: F responses; Table S7: Neurophysiological Investigations: Near-nerve technique; Table S8: Neurophysiological Investigations: EMG MUP Data; Figure S1: (A–C) Sensory responses. (D–F) Recordings obtained using the near-nerve technique. (A) Left Median nerve; (B) Left Ulnar nerve; (C) Right Ulnar nerve; (D) Right Median nerve; (E) Left Sural nerve; (F) Right Sural nerve. Recording parameters: Sensitivity (µV/D); Sweep (ms/D); Averaging (Med). Reference [54] was cited in the supplementary materials.

## Abbreviations

The following abbreviations are used in this manuscript:

WNK1	Protein kinase, lysine-deficient 1
HSAN(s)	Hereditary sensory and autonomic neuropathy (neuropathies)
HSAN 1	Hereditary sensory and autonomic neuropathy type 1
HSAN 2	Hereditary sensory and autonomic neuropathy type 2
HSAN 3	Hereditary sensory and autonomic neuropathy type 3
HSAN 4	Hereditary sensory and autonomic neuropathy type 4
HSAN 5	Hereditary sensory and autonomic neuropathy type 5
HSAN 6	Hereditary sensory and autonomic neuropathy type 6
HSAN 7	Hereditary sensory and autonomic neuropathy type 7
HSAN 8	Hereditary sensory and autonomic neuropathy type 8
HSAN 9	Hereditary sensory and autonomic neuropathy type 9
HSAN 2A	Hereditary sensory and autonomic neuropathy type 2A
HSAN 2D	Hereditary sensory and autonomic neuropathy type 2D
TECPR2	Tectonin beta-propeller repeat-containing protein 2
HSP 49	Hereditary spastic paraplegia 49
IKBKAP/ELP1	Inhibitor of kappa light polypeptide gene enhancer in B cells, kinase complex-associated protein/ Elongator protein 1
SPTLC1	Serine palmitoyltransferase, long-chain base subunit 1
SPTLC2	Serine palmitoyltransferase, long-chain base subunit 2
RAB7A	Ras-Related Protein Rab-7A
ATL1	Atlastin GTPase 1
DNMT1	DNA methyltransferase 1
NTRK1	Neurotrophic tyrosine kinase receptor type 1
NGFB	Nerve growth factor beta
DST	Dystonin
SCN11A	Sodium voltage-gated channel alpha subunit 11
SCN9A	Sodium voltage-gated channel alpha subunit 9
KIF1A	Kinesin Family Member 1A
RETREG1 (FAM134B)	Reticulophagy regulator 1 (Family with Sequence Similarity 134, Member B)
MFN2	Mitofusin 2
NEFL	Neurofilament protein, light polypeptide
GDAP1	Ganglioside-induced differentiation-associated protein 1
AD	Autosomal dominant
AR	Autosomal recessive
CMT2	Charcot–Marie–Tooth neuropathy type 2
EMG	Electromyography
NCS	Nerve conduction studies
SNAPs	Sensory nerve action potentials
CMAP	Compound muscle action potential
DRG	Dorsal root ganglia
SNHL	Sensorineural hearing loss
TRPV4	Transient receptor potential cation channel subfamily V member 4
OH	Orthostatic hypotension
GERD	Gastroesophageal reflux disease
TF	Thermoregulatory failure
