# Supplementary material for: Hereditary Sensory and Autonomic Neuropathy Type 2: A Case Report and a Review of the Literature
Source: Brain Sci. 2025 Oct 29;15(11):1163. doi: 10.3390/brainsci15111163 (PMC12650649; doi:10.3390/brainsci15111163)
Supplement: Supplementary file 1 [file brainsci-15-01163-s001.zip › brainsci-3857012-supplementary.pdf]

SUPPLEMENTARY MATERIALS

Category	Test/Investigation
Autoimmune screening	Serum immunofixation, Serum electrophoresis, Free lambda chains ANA, ANCA, Anti-cardiolipin IgM and IgG, Anti-beta2 glycoprotein I IgM and IgG antibodies, ESR, Cryoglobulins
Microbiological screening	HIV, HCV, HBV, Anti-Treponema pallidum IgM and IgG antibodies
Anti-neuronal antibodies	Anti-amphiphysin, anti-CV2, anti-PNMA2, anti-Ri, anti-Yo, DNER, Recoverine, SOX1, Titin, Zic4, GAD65, anti-Hu
Anti-gangliosides antibodies IgG and IgM	Anti-GM1, GM2, GM3, GD1a GD1b, GT1b, GQ1b
Autoimmune encephalitis panel	Anti-NMDA, anti-CASPR2, anti-AMPA 1/2, anti-LGI1, anti-DPPX, anti-GABAb

Supplementary Table S1. Laboratory investigations.

Keys: ANA: Antinuclear antibodies; ANCA: Anti-neutrophil cytoplasmic antibodies; ESR: Erythrocyte sedimentation rate

Hereditary neuropathies panel	Methods
AARS, ABCA1, ABHD12, ACOX1, AGTPBP1, AGXT, AIFM1, APOA1, APTX, ARSA, ATL1, ATM, ATP1A1, ATP7A, B4GALNT1, BAG3, BCKDHB, BICD2, BSCL2, C12orf65, C1orf194, CADM3, CD59, CHCHD10, CNTNAP1, COA7, COQ7, COX20, COX6A1, CPOX, CTDP1, CYP27A1, DARS2, DCTN1, DEGS1, DHTKD1, DHX9, DNAJB2, DNAJC3, DNM2, DNMT1, DRP2, DST, DYNC1H1, EGR2, ELP1, ERCC6, ERCC8, FAH, FAM126A, FBLN5, FGD4, FIG4, FLVCR1, FXN, GALC, GAN, GARS, GBA2, GBF1, GDAP1, GJB1, GJC2, GLA, GNB4, GSN, HADHA, HADHB, HARS, HEXA, HEXB, HINT1, HK1, HMBS, HSPB1, HSPB8, IARS2, IGHMBP2, INF2, ITPR3, KCNA2, KIF1A, KIF1B, KIF5A, LITAF, LMNA, LRSAM1, LYST, MAG, MCM3AP, MFN2, MMACHC, MME, MORC2, MPV17, MPZ, MTMR2, MTTP, MYH14, NAGA, NDC1, NDRG1, NEFH, NEFL, NEMF, NGF, NTRK1, OPA1, OPA3, PDHA1, PDK3, PDXK, PDYN, PEX10, PEX7, PHYH, PIGB, PLEKHG5, PLP1, PMM2, PMP2, PMP22, PNKP, PNPLA6, POLG, POLR3A, POLR3B, PPOX, PRDM12, PRNP, PRPS1, PRX, PTPN11, RAB7A, REEP1, RETREG1, RTN2, SACS, SARS, SBF1, SBF2, SCARB2, SCN10A, SCN11A, SCN9A, SCO2, SEPT9, SETX, SH3TC2, SIGMAR1, SLC12A6, SLC25A19, SLC25A46, SLC52A2, SLC52A3, SLC5A6, SLC5A7, SMN1, SORD, SOX10, SPAST, SPG7, SPG11, SPTAN1, SPTBN4, SPTLC1, SPTLC2, SURF1, SYT2, TECPR2, TFG, TRIM2, TRPA1, TRPV4, TTPA, TTR, TUBB3, TYMP, UBA1, VAPB, VCP, VPS13A, VRK1, VWA1, WARS, WNK1, XK, XPA, YARS, ZFYVE26	**Target:** coding genomic regions and exon–intron junctions (±100 bp)<br>**Method used:** library preparation by enzymatic fragmentation of DNA (xGen™ DNA EZ Library Prep Kit, IDT), enrichment with probes (xGen Exome Research Panel v2, IDT), and paired-end 2×100 sequencing on the NovaSeq 6000 platform (Illumina). Validation performed by PCR amplification*, sequencing reaction, and capillary electrophoresis run using the Sanger method (ABI 3500dx).<br>**Reference databases and software supporting variant interpretation:** ClinVar, gnomAD v4.1.0, PubMed, Varsome, Franklin, PanelApp.

Supplementary Table S2. In silico genes panel analysis for hereditary neuropathies panel.

**Validation performed by Sanger sequencing of Likely Pathogenic (Class 4) and Pathogenic (Class 5) variants.*

Primary antibodies	Secondary antibodies
PGP9.5 (pan-neuronal marker)	Cyanine 3
CollIV (collagen marker)	Alexa fluor 488
**Leg**	
Epidermal innervation: Absent (3 sections quantification), reference cutoff 10,3 [54]	
Sweat gland innervation: low density; morphological abnormalities	
Erector pili muscle innervation: low density; morphological abnormalities	
**Thigh**	
Epidermal innervation: Absent (3 sections quantification), reference cutoff 14,5°	
Sweat gland innervation: low density; morphological abnormalities	
Erector pili muscle innervation: low density; morphological abnormalities	

Supplementary Table S3. Histopathological investigations: Protocol and Results.

The skin biopsy was performed at distal leg (10 cm proximal to lateral malleolus) and thigh (10 cm above the knee). The analysis was performed via indirect immunofluorescence (PGP 9.5, Collagen IV staining).

°Laboratory reference cutoff

Nerve	Lat	Amp	CV	Dist	Tot. Duration	Tot. Area	Neg. Amp. Diff. %
	ms	mV	m/s	mm	ms	ms*mV	%
**Left Median Motor**							
Wrist-ABP | APB	3.39	7.1		70.0			
Elbow-Wrist | APB	7.28	7.0	51.7	201			-1.41
Axilla-Elbow | APB	9.23	6.9	57.9	113			-1.43
**Right Median Motor**							
Wrist-ABP | APB	3.37	8.5		70.0			
Elbow-Wrist | APB	7.93	7.8	48.5	221			-8.2
Axilla-Elbow | APB	9.82	7.7	54.0	102			-1.28
Erb-Axilla | APB	3.60	5.0	--				-35.1
**Left Tibial Motor**							
Ankle-Abd hal | Abd hal	3.27	12.1		90.0			
Knee-Ankle | Abd hal	11.6	7.1	45.3	377			-41.3
**Right Tibial Motor**							
Ankle-Abd hal | Abd hal	3.12	14.6		83.0			
Knee-Ankle | Abd hal	11.1	10.2	47.5	379			-30.1
**Left Ulnar Motor**							
Wrist-ADM | ADM	2.66	10.4		70.0			
Bl. elbow-Wrist | ADM	6.35	10.0	49.3	182			-3.8
Ab. elbow-Bl. elbow | ADM	8.59	9.7	46.9	105			-3.0
Axilla-Ab. Elbow | ADM	10.9	9.5	44.6	103			-2.1
**Right Ulnar Motor**							
Wrist-ADM | ADM	2.90	11.2		70.0			
Bl. elbow-Wrist | ADM	6.63	10.6	48.5	181			-5.4
Ab. elbow-Bl. elbow | ADM	8.75	10.7	50.0	106			0.94
Axilla-Ab. Elbow | ADM	10.7	10.7	56.4	110			0

Supplementary Table S4. Neurophysiological Investigations: Motor Nerves

Keys: Lat: Latency; Amp: Amplitude; CV: Conduction Velocity; Dist: Distance; Tot. Duration: Total Duration; Tot. Area: Total Area; Neg. Amp. Diff. %: Negative amplitude difference percentage; ABP: Abductor pollicis brevis muscle; Bl. elbow: Below elbow; Ab. elbow: Above elbow; ADM: Abductor digiti minimi muscle.

Nerve	Lat	Amp 1-2	Amp 2-3	CV	Dist
	ms	uV	uV	m/s	mm
**Left Median Sensory**					
Wrist-Dig II	12.0	9.1		10.9	131
**Left Ulnar Sensory**					
Wrist-Dig V	4.27	3.0		26.5	113
**Right Ulnar Sensory**					
Wrist-Dig V	--	--			126

Supplementary Table S5. Neurophysiological Investigations: Sensory Nerves

Keys: Lat: Latency; Amp: Amplitude; CV: Conduction Velocity; Dist: Distance; Dig II: Second Digit; Dig V: Fifth Digit.

Nerve	F Lat (Mean)	% F
	ms	%
**Left median F-wave response**		
Wrist-APB	30.9	
**Right median F-wave response**		
Wrist-APB	29.6	
**Left tibial F-wave response**		
Ankle-Abd hal	48.9	
**Right tibial F-wave response**		
Ankle-Abd hal	49.7	
**Left ulnar F-wave response**		
Wrist-ADM	30.1	
**Right ulnar F-wave response**		
Wrist-ADM	29.5	

Supplementary Table S6. Neurophysiological Investigations: F responses

Keys: F Lat (Mean): Mean F-wave latency; % F: F-wave persistence (%); ABP: Abductor pollicis brevis muscle; ADM: Abductor digiti minimi muscle.

Nerve	Lat	Amp	CV	Dist	Stim Int
	ms	uV	m/s	mm	mA
**Right Median nerve**					
Dig I-Wrist	--	--		100	31.6
Dig III-Wrist	--	--		135	23.8
**Left Sural nerve**					
Lat. Mall.-Calf	--	--		107	2.7
**Right Sural nerve**					
Lat. Mall.-Calf	--	--		110	5.8

Supplementary Table S7. Neurophysiological Investigations: Near-nerve technique

Keys: Lat: Latency; Amp: Amplitude; CV: Conduction Velocity; Dist: Distance; Stim Int: Stimulus Intensity; Dig I: First Digit; Dig III: Third Digit; Lat. Mall: Lateral Malleolus.

Muscle	Mean Amplitude	Mean Duration	% Poly
	uV	ms	%
Right tibialis anterior muscle	496	14.4	15.0

Supplementary Table S8. Neurophysiological Investigations: EMG MUP Data

Keys: % Poly: Percentage of polyphasic potentials.

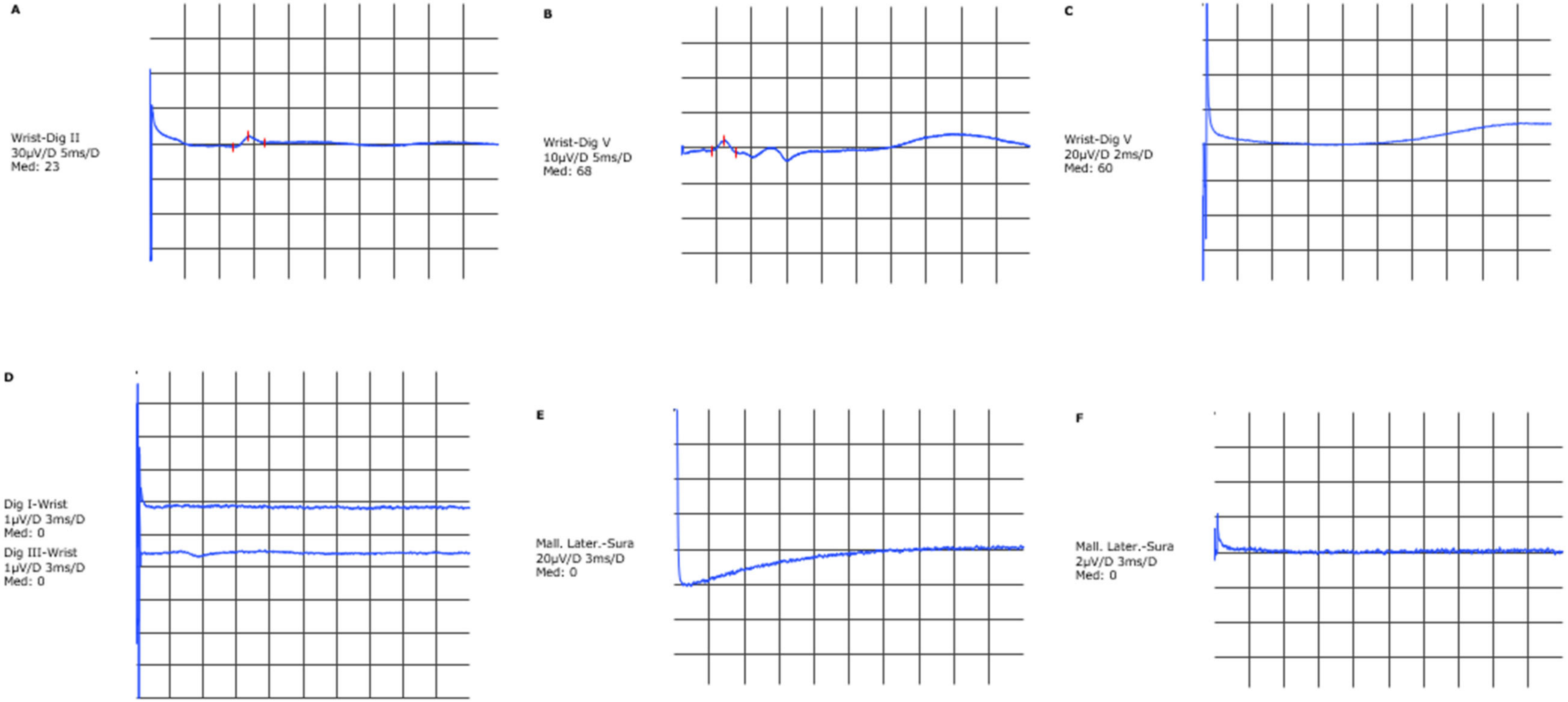

Supplementary Figure S1. (A–C) Sensory responses. (D–F) Recordings obtained using the near-nerve technique. (A) Left Median nerve; (B) Left Ulnar nerve; (C) Right Ulnar nerve; (D) Right Median nerve; (E) Left Sural nerve; (F) Right Sural nerve. Recording parameters: Sensitivity (μV/D); Sweep (ms/D); Averaging (Med).
